# Gli1 Transcriptional Activity is Negatively Regulated by AKT2 in Neuroblastoma

**DOI:** 10.18632/oncotarget.1074

**Published:** 2013-06-29

**Authors:** Pritha Paul, Natasha Volny, Sora Lee, Jingbo Qiao, Dai H. Chung

**Affiliations:** ^1^ Department of Pediatric Surgery, Vanderbilt University Medical Center, Nashville, TN; ^2^ Department of Cancer Biology, Vanderbilt University Medical Center, Nashville, TN

**Keywords:** Gli1, AKT2, Neuroblastoma, SUFU

## Abstract

Activation of the Hedgehog (Hh) signaling pathway has been implicated in a variety of malignancies including neuroblastoma. Expression of Gli1, a downstream effector of Hh, correlates with a favorable prognosis in patients with neuroblastoma. Moreover, Gli1 overexpression reduces mitotic index and induces transcription of genes involved in the differentiation of neuroblastoma cells; however, much remains unknown regarding the regulation of Gli1 transcriptional activity. Here, we report a novel negative regulation of Gli1 transcriptional activity by PI3K/AKT2 signal transduction pathway. Constitutively active PI3K subunit, p110α, inhibited Gli1 transcriptional activity in neuroblastoma cells, whereas, overexpression of an inactive form of PI3K subunit, p85, enhanced its activity. Specifically, the AKT2 isoform inhibited Gli1 luciferase activity. Silencing AKT2 using siRNA increased Gli1 transcriptional activity and conversely, overexpression of constitutively active AKT2 (myr-AKT2) decreased Gli1 transcriptional activity. Furthermore, Gli1 overexpression-mediated decrease in anchorage-independent growth was rescued by AKT2 overexpression. We also demonstrated that AKT2 overexpression regulates the nuclear-cytoplasmic distribution of exogenous Gli1 protein in neuroblastoma cells by relieving a GSK3β-mediated destabilization of SUFU, a negative regulator of Gli1 nuclear translocation. Inhibition of nuclear Gli1 accumulation may explain for the suppression of the tumor-suppressive function of Gli1. Collectively, our findings suggest an important role of Gli1 as a tumor suppressor in neuroblastoma, and offer a mechanism by which AKT2 regulates the subcellular localization, and in turn, inhibits the tumor-suppressive function of Gli1 in neuroblastoma.

## INTRODUCTION

Neuroblastoma arises from the neural crest elements of the sympathetic nervous system. As a pediatric neuroendocrine tumor, studying embryonic developmental pathways involved in the fetal nervous system is warranted. Aberrant activation of one such embryonic pathway, Hedgehog (Hh) signaling, is associated with a variety of cancers [1]. Canonical Hh signaling regulates target gene expression by activating transcription factors, namely Gli1, Gli2 and Gli3 [2]. Interestingly, activation of Hh/Gli signaling has been found in early-stage tumors, and indicates favorable prognosis in neuroblastoma patients [3, 4]. Moreover, Gli1 overexpression reduces the mitotic index of neuroblastoma cells, suggesting a tumor-suppressive role of Gli1 [3]. However, the exact cellular mechanism by which Gli1 exerts its anti-tumorigenic function in neuroblastoma remains unclear.

Since Gli1 is a transcription factor, it is critical to examine upstream signaling pathways that regulate Gli1. Moreover, nuclear accumulation of Gli1 is a pre-requisite for the induction of Gli1-responsive target genes. AKT positively regulates transcriptional activity of sonic hedgehog (Shh)-induced activity of Gli transcription factors in NIH3T3 cells [5]. Also, AKT stabilizes Gli2 and Gli3 by inhibiting their proteolytic processing via protein kinase A (PKA)/casein kinase-1 (CK-1)/glycogen synthase kinase-3β (GSK-3β) [5]. Interestingly, Gli1 acts only as a transcriptional activator and does not undergo proteolytic processing as for Gli2 and Gli3 [6], allowing AKT pathway dispensable for Gli1 signaling. Surprisingly, inhibition of PI3K pathway partially increases Gli1 transcriptional activity in pancreatic cancer cells [7]. Activation of PI3K/AKT signal transduction pathway indicates unfavorable outcomes in neuroblastoma patients [8] and is critical for neuroblastoma cell survival, growth and tumor progression *in vivo* [9]. Recently, isoform based studies of AKT have gained importance as the functional differences among them are becoming increasingly apparent. AKT1 plays a critical role primarily in cell survival and proliferation in cancers, while AKT2 isoform has been implicated in tissue invasion and metastasis [10, 11]. Crosstalk between PI3K/AKT signaling and Gli1 transcriptional activity and thereby, induction of Gli1 downstream targets, may act as a critical determinant in favor of or against Gli1-mediated cellular differentiation in neuroblastoma.

Here, we demonstrate, for the first time, that Gli1 transcriptional activity is negatively regulated by PI3K pathway in neuroblastoma. Furthermore, we provide evidence that the AKT2 isoform, in particular, negatively regulated Gli1 activity. Moreover, Gli1 overexpression inhibited anchorage-independent cell growth; this was rescued by AKT2 overexpression. AKT2-mediated inhibition of Gli1 transcriptional activity suppressed the induction of Gli1 target genes involved in the differentiation of neuroblastoma cells. We also show a novel evidence that AKT2 negatively regulates Gli1 nuclear accumulation, thereby, affecting its function as a transcription modulator in neuroblastoma cells. Interestingly, AKT2 regulated Gli1 subcellular distribution by relieving GSK3β-mediated destabilization of SUFU, a negative regulator of Gli1.

## RESULTS

### PI3K/AKT2 signaling negatively regulated Gli1 transcriptional activity

**Figure 1:**
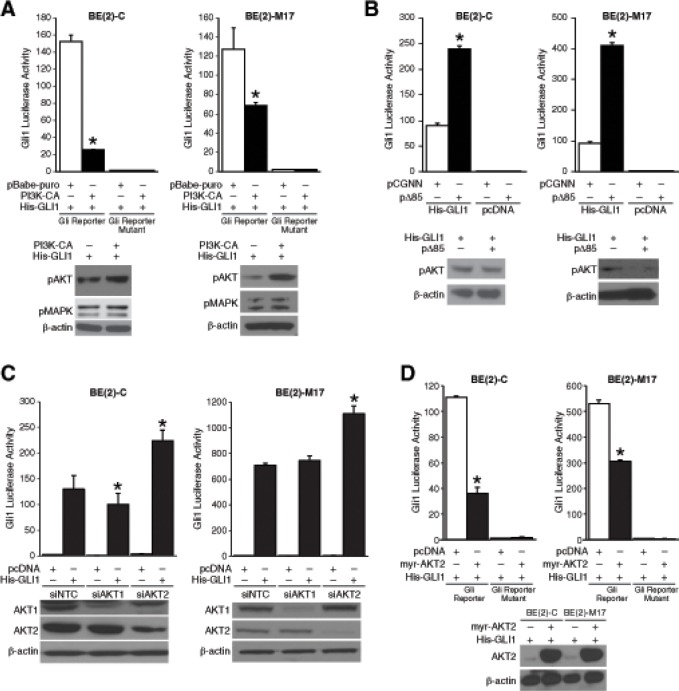
PI3K/AKT2 pathway regulation of Gli1 transcriptional activity (A) BE(2)-C and BE(2)-M17 cells expressing constitutively active form of PI3K subunit, p110α (pBabe-puro-PI3K-CA), decreased Gli1 transcriptional activity compared to cells transfected with vector control (pBabe-puro). Expressions of pAKT and pMAPK were used as indicators of activation of PI3K and MAPK pathways, respectively. (B) Inhibition of PI3K using an inactive form of PI3K subunit, p85 (pCGNN-Δp85), increased Gli1 transcriptional activity in comparison to pCGNN control cells. (C) Silencing of AKT2, but not AKT1, increased Gli1 transcriptional activity transiently expressing His-GLI1 or pcDNA control. Efficacy of siRNA is demonstrated by immunoblotting. (D) Overexpression of constitutively active form of AKT2 (pcDNA-myr-AKT2) inhibited Gli1 transcriptional activity in comparison to cells transfected with control vector (mean ± SEM; *=*p* < 0.05).

Whereas K-Ras signaling enhances Gli1 transcriptional activity in pancreatic cancer [7], protein kinase C and PKA negatively regulate Gli1 by affecting its nuclear localization in hepatocellular carcinoma and NIH3T3 cells, respectively [12, 13]. AKT signaling is necessary for Gli transcriptional activity in NIH3T3 cells [5]. Interestingly, inhibition of PI3K pathway by wortmannin partially increased Gli1 luciferase activity without affecting *GLI1* mRNA levels in pancreatic cancer cells [7]. Here, we sought to examine this apparent negative regulation of Gli1 transcriptional activity by PI3K, an oncogenic signaling pathway critical for neuroblastoma progression [14]. Using a robust luciferase reporter system (Supplemental Fig. S1), we sought to examine the regulation of Gli1 transcriptional activity by PI3K pathway. Gli1 luciferase activity decreased by ~75% and ~ 50% in p110α (PI3K-CA) overexpressing BE(2)-C and BE(2)-M17 cells, respectively, in comparison to cells transfected with pBabe-puro control vector (Fig. 1A). Overexpression of Δp85 (containing a deletion mutation in the PI3K regulatory subunit p85) increased Gli1 transcriptional activity by ~3 folds compared to vector control cells (Fig. 1B). Interestingly, silencing AKT2, but not the AKT1 isoform, significantly increased Gli1 luciferase activity in both human neuroblastoma cell lines examined, thus demonstrating negative regulation of Gli1 by AKT2 (Fig. 1C). Conversely, overexpression of AKT2 suppressed Gli1 transcriptional activity in BE(2)-C and BE(2)-M17 cells by approximately 67% and 40%, respectively, when compared to pcDNA controls (Fig. 1D). Collectively, our data suggest an inhibitory role for the PI3K/AKT2 signaling on Gli1 transcriptional activity in neuroblastoma cells.

### Gli1 overexpression reduced anchorage-independent growth

**Figure 2:**
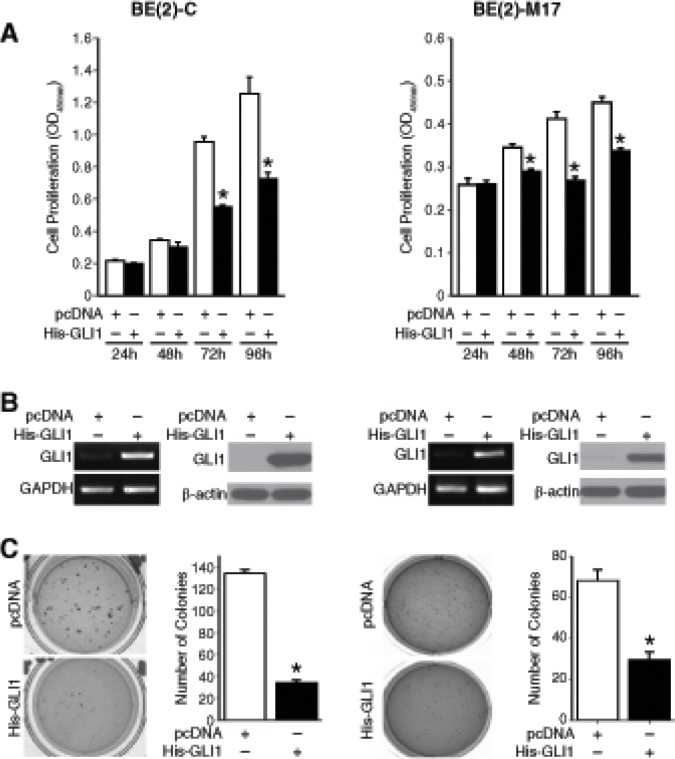
Anti-tumor function of Gli1 in neuroblastoma (A) Gli1 overexpression inhibited neuroblastoma cell proliferation. (B) *GLI1* mRNA and Gli1 protein were confirmed by semi-quantitative PCR and immunoblotting, respectively. (C) Anchorage-independent growth was assessed after transfection with pcDNA control vector or His-GLI1. Overexpression of *Gli1* inhibited anchorage-independent growth as assessed by the number of colonies formed. (mean ± SEM; *=*p* < 0.05).

Gli1 activity is generally associated with disease progression in cancer [1]. Hence, we targeted *GLI1* using siRNA and examined the proliferative capacity of multiple human neuroblastoma cells lines (Supplemental Fig. S2A). There was no significant change in the proliferation rate of multiple human neuroblastoma cell lines tested, though there were slight trends toward growth stimulatory effects in all cell lines except SK-N-DZ. Downregulation of *GLI1* mRNA level was confirmed by real-time PCR (Supplemental Fig. S2B). Similarly, cyclopamine, a specific antagonist of the hedgehog signaling pathway, failed to inhibit neuroblastoma cell growth *in vitro* (Supplemental Fig. S2C). Hh signaling perturbation by cyclopamine was confirmed by checking *GLI1* mRNA levels (Supplemental Fig. S2D). This was not surprising as Gli1 overexpression induces differentiation of SH-SY5Y neuroblastoma cells and decreases the rate of mitosis in a number of *MYCN*-amplified neuroblastoma cell lines *in vitro* [3]. A similar decrease in cell proliferation was observed in both BE(2)-C and BE(2)-M17 cell lines after Gli1 overexpression (Fig. 2A). Overexpression was confirmed by analyzing *GLI1* mRNA and protein levels using semi-quantitative real-time PCR and immnuoblotting, respectively (Fig. 2B). Anchorage-independent growth on a semi-solid substratum reflects the tumorigenicity of cancer cells *in vitro*. Overexpression of pcDNA-His-GLI1 in BE(2)-C cells reduced tumorigenicity *in vitro* as evident from an approximately 80% decrease in the number of soft agar colonies formed as compared to controls (Fig. 2C). BE(2)-M17 cells overexpressing Gli1 demonstrated ~ 60% decrease in colony formation when compared to controls (Fig. 2C). Our data confirm the tumor-suppressive role of Gli1 in neuroblastoma as opposed to its oncogenic potential in basal cell carcinoma and medulloblastoma.

### AKT2 inhibited the tumor-suppressive role of Gli1 in neuroblastoma cells

**Figure 3:**
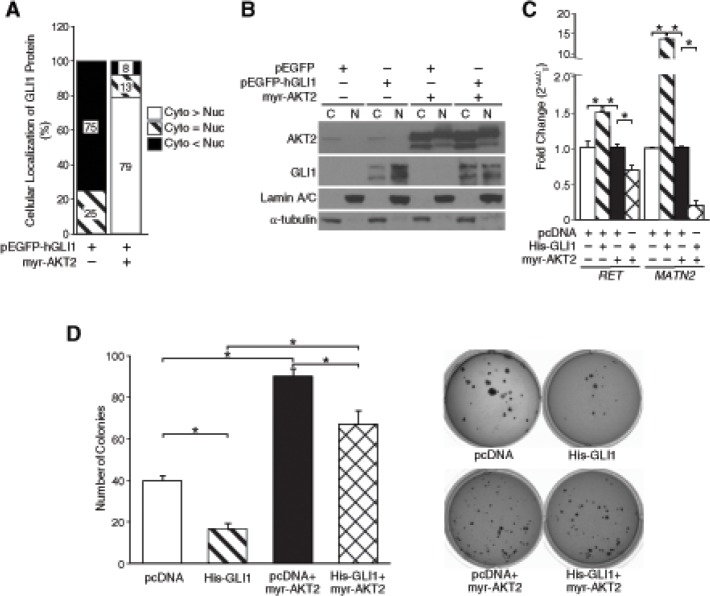
AKT2 regulated Gli1 function by modulating its nuclear-cytoplasmic shuttling (A) The percentage of Gli1-GFP in each cellular compartment was calculated from images taken from multiple fields of view (Supplemental Fig. S3). Cyto > Nuc indicates preferential nuclear localization, Cyto = Nuc indicates distribution in both nucleus and cytoplasm, Cyto > Nuc indicates predominantly cytoplasmic localization. (B) Cell fractionation studies indicate subcellular distribution of pEGFP-hGLI1 and AKT2 with or without overexpression of myr-AKT2 in BE(2)-C cells. α-tubulin was used as a control for cytoplasmic fraction and Lamin A/C for the nuclear fraction. (C) mRNA levels of Gli1 downstream targets, *RET* and *MATN2*, were quantified to assess transcriptional activity of Gli1 with or without AKT2 overexpression. (D) Anchorage-independent growth was assessed in BE(2)-C cells after transfection with pcDNA control vector alone, His-GLI1 alone, myr-AKT2 alone or co-transfection of His-GLI1 and myr-AKT2. Overexpression of myr-AKT2 increased anchorage-independent growth when compared to vector control and rescued suppression of soft-agar colony formation by Gli1 overexpression (mean ± SEM; *=*p* < 0.05).

Nuclear localization and accumulation of Gli1 is necessary for exhibiting its transcriptional activity. Using fluorescence imaging, we observed that pEGFP-hGLI1 was localized primarily in the nucleus of BE(2)-C cells when compared to pEGFP control cells where fluorescence was observed diffusely both in the cytoplasm and nucleus (Supplemental Fig. S3). Although, AKT2 overexpression had no specific effects on the distribution of GFP signal in BE(2)-C cells transfected with pEGFP only, it retained Gli1-GFP in the cytoplasm (~79%) of BE(2)-C cells when compared to pEGFP-hGLI1 alone (~5%) (Fig. 3A). We further confirmed the distribution of His-GLI1, endogenous AKT2 and myr-AKT2 between the cytoplasm and nucleus by cell fractionation and immunoblotting. Expression of endogenous AKT2 was predominantly in the cytoplasm, whereas, myr-AKT2 is expressed at a similar level in both the cytoplasm and nucleus of BE(2)-C cells. Both endogenous and exogenous AKT2 expression remained unaffected by Gli1 overexpression (Fig. 3B). Overexpression of His-GLI1 alone indicated a preferential nuclear expression of Gli1 in BE(2)-C cells. AKT2 overexpression decreased the nuclear accumulation of exogenous Gli1 (Fig. 3B). This was in accordance to the GFP images (Supplemental Fig. S3) and indicated cytoplasmic retention of Gli1 after AKT2 overexpression. Our findings identify a potential novel role for AKT2 in the cytoplasmic retention of Gli1 in neuroblastoma cells.

To determine the functional significance of AKT2-mediated inhibition of Gli1 transcriptional activity in neuroblastoma, we quantified the mRNA levels of *RET* and *MATN2* as downstream targets of Gli1 [3] with or without AKT2 overexpression. AKT2 overexpression significantly suppressed Gli1-mediated transcription of *RET* and *MATN2* in BE(2)-C cells (Fig. 3C). Moreover, AKT2 overexpression significantly increased anchorage-independent growth of BE(2)-C cells when compared to cells transfected with vector control, thus further demonstrating its role as an oncogene in neuroblastoma (Fig. 3D). Interestingly, overexpression of myr-AKT2 rescued the suppression of anchorage-independent growth upon Gli1 overexpression in BE(2)-C cells in comparison to Gli1 overexpression alone (Fig. 3D). Taken together, our data confirms that AKT2 inhibits the tumor-suppressive role of Gli1 by suppressing its transcriptional activity in neuroblastoma cells.

### AKT2 regulated nuclear-cytoplasmic shuttling of Gli1 protein

**Figure 4:**
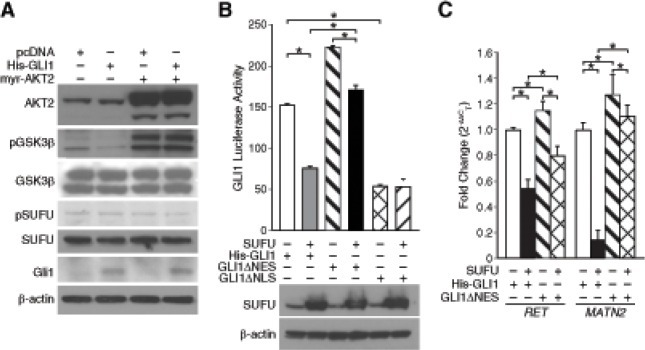
AKT2 relieved GSK3β-mediated inhibition of SUFU and retained Gli1 in the cytoplasm of neuroblastoma cells (A) Immunoblotting confirmed overexpression of Gli1 and AKT2, and enhanced phosphorylation of GSK3β after AKT2 overexpression. Expression of pSUFU was decreased after AKT2 overexpression. β-actin confirms equal loading. (B) SUFU overexpression decreased luciferase activity of His-GLI1 to an extent more than the GLI1ΔNES form. GLI1ΔNLS had a lower luciferase activity in comparison to both wild-type His-GLI1 and GLI1ΔNLS. (C) Transcription of Gli1 target genes, *RET* and *MATN2*, was higher when BE(2)-C cells were transfected with GLI1ΔNES compared to His-GLI1. SUFU overexpression inhibited transcription of target genes by His-GLI1 to a greater extent in comparison to GLI1ΔNES-mediated transcription (mean ± SEM; *=*p* < 0.05).

We next sought to identify a mechanism by which AKT2 might negatively regulate Gli1 subcellular localization and thereby nuclear translocation. AKT antagonizes GSK3β signaling by direct phosphorylation. GSK3β in conjunction with PKA and CK-1 regulate the proteolytic degradation of Gli2 and Gli3 [5]. Interestingly, phosphorylation of GSK3β stabilizes suppressor-of-fused (SUFU) under ligand-independent conditions [15] and SUFU in turn negatively regulates Gli1 nuclear translocation [16]. To examine the possibility that AKT2 may decrease Gli1 activity by relieving GSK3β-mediated destabilization of SUFU, we assessed the expression of pGSK3β and SUFU after AKT2 overexpression. AKT2 overexpression increased phosphorylation of GSK3β in BE(2)-C cells transfected either with vector control of pcDNA-His-GLI1 (Fig. 4A). However, there was no appreciable difference observed in the expression of endogenous SUFU with or without AKT2 overexpression (Fig. 4A). This observation is not surprising as it is the phosphorylation status and subsequent degradation of SUFU, which impacts Gli1 transcriptional activity by allowing nuclear translocation of Gli1. The levels of phosphorylated SUFU was decreased after AKT2 overexpression in BE(2)-C cells (Fig. 4A), indicating a possibility that AKT2 via SUFU negatively regulated Gli1 transcriptional activity in neuroblastoma cells.

To confirm the inhibitory effect of SUFU on Gli1 transcriptional activity based on Gli1 localization, we used two constructs GLI1ΔNES and GLI1ΔNLS [13]. Compared to wild-type Gli1 (His-GLI1), GLI1ΔNES localizes primarily to the nucleus due to a defective nuclear export signal, whereas, GLI1ΔNLS is predominantly cytoplasmic because of lack of an effective nuclear localization signal [13]. Our luciferase data showed that transcriptional activity of His-GLI was significantly decreased (~50%) when co-expressed with HA-SUFU in BE(2)-C cells in comparison to His-GLI1 alone (Fig. 4B). This confirms the negative role of SUFU in Gli1 transcriptional activity as previously demonstrated [16] and a novel demonstration of the same in neuroblastoma. GLI1ΔNES demonstrated a higher luciferase activity compared to His-GLI1 and overexpression of SUFU decreased the luciferase activity of GLI1ΔNES by ~23% (Fig. 4B). Interestingly, an approximate 50% decrease in Gli1 transcriptional activity by co-expression of SUFU is less compared to the AKT2-mediated inhibition of Gli1 transcriptional activity (~67%, Fig. 1D). This striking observation indicated that SUFU-dependent nuclear export of Gli1 only partially explains AKT2-mediated regulation of Gli1 subcellular localization and potential alternative mechanisms exist by which AKT2 might exert its negative regulatory effect on Gli1 transcriptional activity. As expected, SUFU overexpression did not significantly alter the low luciferase activity of GLI1ΔNLS (Fig. 4B). Real-time PCR was used to confirm the inhibitory role of SUFU on the transcription of Gli1 target genes, *RET* and *MATN2* (Fig. 4C).

## DISCUSSION

Gli1 is necessary and sufficient for disease progression in basal cell carcinoma and medulloblastoma [17, 18]. In this respect, neuroblastoma differs from these cancers as Gli1 expression correlates with a more favorable prognosis in patients and induces differentiation of neuroblastoma cells [3, 4]. Moreover, Gli1 overexpression has been shown to decrease the mitotic index of *MYCN*-amplified neuroblastoma cells [3]. Our data provides evidence, for the first time, that overexpression of Gli1 retards proliferation and reduces anchorage-independent growth of *MYCN*-amplified neuroblastoma cells. The slower growth rate of neuroblastoma cells overexpressing Gli1 may be due to reduced cell proliferation as no appreciable change in apoptosis was observed (data not shown).

Gli1 functions as a nuclear transcription factor and multiple signaling pathways regulate its activity. Similar to previous studies [7], our results indicated that BRAF/MEK/ERK signaling positively regulates Gli1 transcriptional activity in neuroblastoma cells (data not shown). AKT signaling is necessary for Gli transcriptional activity in NIH3T3 cells [5], but dispensable in pancreatic cancer and keratinocytes [7, 19]. Here, we report a PI3K/AKT2-dependent negative regulation of Gli1 transcriptional activity by AKT2 in neuroblastoma cells. Previous studies employed either less specific pharmacologic PI3K inhibitors or targeted AKT1 alone to examine the regulation of Gli1 by the PI3K/AKT pathway. By modulating the expression of specific genes in the PI3K signaling, we found that PI3K inhibits Gli1 transcriptional activity in neuroblastoma cells. This is a novel observation demonstrating a potential role for PI3K-subunits, p110 and p85, in the regulation of Gli1 function. PI3K signaling activates multiple signaling cascades apart from the classical downstream effector, AKT. It is important to examine the role of non-classical effectors of PI3K signaling in the regulation of Gli1 function. Sonic hedgehog (Shh) treatment stimulates fibroblast migration by PI3K-dependent activation of small Rho GTPases Rac1 and RhoA, but this phenomenon occurs independent of Gli1 signaling [20]. Much is unknown about how Gli1 signaling is regulated in neuroblastoma. For our study, we focused mainly on the AKT isoforms that are well known for their role in cell proliferation and evasion of apoptosis.

Activation of PI3K/AKT signal transduction pathway is a marker for disease progression in neuroblastoma patients [8]. We have previously demonstrated the critical role of PI3K/AKT signaling in angiogenesis and metastasis [9, 21]. Recently, isoform based studies of AKT have gained importance as the functional differences amongst the isoforms are becoming increasingly apparent. AKT1 primarily plays a critical role in cell survival and proliferation in multiple cancers, while AKT2 isoform has been implicated in tissue invasion and metastasis [10, 11, 22-24]. Inactivation of AKT decreases Gli1 and Gli2 transcriptional activity in NIH3T3 cells, and silencing AKT1 inhibited Shh-mediated activation of Hh reporter [5]. Our data does not dispute the possibility of AKT1 as a positive regulator of Gli1 transcriptional activity, especially, in BE(2)-C cell line but not in BE(2)-M17. Hence, the effects of AKT1 isoform on the regulation of Gli1 may be cell line specific and thus, require future studies for clarification. Surprisingly, the AKT2 isoform negatively regulated Gli1 function in both neuroblastoma cell lines we tested. This is consistent with the observation that overexpression of constitutively active AKT2 not only failed to rescue Gli1 activity in pancreatic cancer cells lacking K-Ras, but also further decreased it, albeit not significantly [7]. Moreover, we demonstrated that AKT2 overexpression relieved Gli1-mediated suppression of anchorage-independent growth of neuroblastoma cells and downregulated the transcription of Gli1 target genes in neuroblastoma cells, indicating that AKT2 potentially regulated the function of Gli1 as a transcription factor.

AKT-mediated phosphorylation inhibits the function of transcription factors, such as FOX and FOXOs, involved in embryonic development, differentiation, and cell cycle arrest, by regulating nuclear-cytoplasmic shuttling, and in turn, stabilization of these proteins [25, 26]. One of the negative regulators of AKT is GSK3β. Recently, a novel role has been described for GSK3β in regulating the function of SUFU as an inhibitor of Gli transcription factors [15] and may explain the inhibitory role of AKT2 on Gli1 transcriptional activity. Our observations not only indicates data similar to previous studies describing co-localization of overexpressed SUFU with Gli1 in the nucleus and consequent inhibition of the transcription of Gli1 target genes [16, 27], but also implicates AKT2/GSK3β in regulating SUFU-dependent Gli1 subcellular localization, and subsequently its function as a transcription factor.

Studies regarding Gli1 in neuroblastoma are limited to immunohistochemical analysis of patient samples and preliminary *in vitro* experiments. *In vivo* studies will be of great significance to address the role of Gli1 in different phases of tumorigenesis. In conclusion, Gli1 transcriptional activity is inhibited by AKT2 via regulation of its subcellular distribution. Targeting AKT2 signaling and/or activating Gli1 signaling may represent a new therapeutic strategy against undifferentiated neuroblastoma that is often refractory to current treatment regimen.

## MATERIAL AND METHODS

### Reagents

Antibodies against Gli1, pMAPK, pAKT (S473), AKT1, AKT2, pGSK3β, GSK3β, Lamin A/C and cell lysis buffer were obtained from Cell Signaling Technology (Beverly, MA). Antibody against SUFU was purchased from Santa Cruz Biotechnology (Santa Cruz, CA). Phospho-SUFU antibody was purchased from Sigma-Aldrich (St. Louis, MO). Anti β-actin antibody and fetal bovine serum (FBS) were from Sigma-Aldrich. NuPAGE Novex 4–12% Bis–Tris Gel and Lipofectamine 2000 were from Invitrogen (Carlsbad, CA). Horseradish peroxidase (HRP)-conjugated secondary antibodies against mouse and rabbit IgG, and α-tubulin antibody were obtained from Santa Cruz Biotechnology. Enhanced chemiluminescence (ECL) HRP substrate was purchased from Millipore (Immobilon Western) and Perkin Elmer (Western Lightning).

### Cell culture, plasmids and transfection

Human neuroblastoma cell lines, BE(2)-C and BE(2)-M17, were purchased from American Type Culture Collection (Manassas, VA). Cells were maintained in RPMI 1640 media with L-glutamine (CellGro Mediatech, Inc. Herndon, VA) supplemented with 10% FBS. Cells were maintained at 37°C in a humidified atmosphere of 95% air and 5% CO_2_. For transfection, cells were plated in 6-well plates and transfected with siRNA (100 nM) or plasmids (total of 4 μg) using Lipofectamine 2000 as per manufacturer's protocol. SMARTPool *AKT1* and *AKT2* siRNA were obtained from Dharmacon, Inc. (Lafayette, CO). pcDNA-His-GLI1, 8X3'GLI-BS-Luc and its mutant form were gifts from Dr. Hiroshi Sasaki (RIKEN, Japan). pBabe-Puro-myr-HA-PI3KCA (12523) was obtained from Addgene (Cambridge, MA). pcDNA-myr-AKT2 was a kind gift from Dr. Mark Evers (University of Kentucky, Lexington). pEGFP-hGLI1, GLI1ΔNES and GLI1ΔNLS were gifts from Dr. Jingwu Xie, (Indiana University Medical Center). pCMV-HA-SUFU was kindly provided by Dr. Chin Chiang (Vanderbilt University Medical Center). For stable transfection of His-GLI1 or pcDNA control vector, cells were selected with G418 (300 μg/mL).

### Luciferase reporter assay

To examine Gli1 transcriptional activity, pcDNA-His-GLI1 (0.5 μg/well), pTK-Renilla control plasmid (20 ng/well) and siRNA (100 nM) or plasmids (1 μg) were co-transfected with the Gli reporter plasmids in 6-well plates using Lipofectamine 2000. After 24 h, the cells were plated in 24-well plates, and incubated in 5% CO_2_ at 37°C overnight. Cells were harvested and luciferase activity was measured with Dual-GLO luciferase reporter assay system (Promega, Madison, WI). Briefly, the transfected cells were lysed in the 24-well plates with 50 μl of reporter lysis buffer, and the lysate was transferred into microcentrifuge tubes. Cell debris was removed by centrifugation for 10 min. Supernatant (20 μl) was mixed with 100 μl of LAR II buffer, and the luminescence was immediately measured (first reading). After 20 sec, 100 μl of Stop & Glo reagent was added to measure the Renilla luciferase activity (second reading). The ratio values from two readings were determined to obtain normalized luciferase activity. Each experiment was repeated three times in triplicate.

### Immunoblotting

Whole-cell lysates were prepared using 1X cell lysis buffer with 1 mM PMSF and incubated on ice for 30–60 min. Total protein (50 μg/lane) was resolved on NuPAGE Novex 4–12% Bis–Tris gels and electrophoretically transferred to polyvinylidene difluoride membranes (Bio-Rad Laboratories, Hercules, CA). Nonspecific binding sites were blocked with 5% milk in TBST (120 mM Tris–HCl, pH 7.4, 150 mM NaCl, and 0.05% Tween 20) for 1 h at room temperature. Target proteins were detected by using rabbit or mouse anti-human antibodies (1:500–2000 dilution) overnight at 4°C. The membranes were washed three times and incubated with secondary antibodies (1:10,000 dilution) conjugated with HRP. Immune complexes were visualized using the ECL system. Equal loading was confirmed with β-actin. Data are representative of three independent experiments.

### Cell proliferation and soft agar colony assay

Human neuroblastoma cells were plated in 96-well plates and cell number was counted after treatments to assess cell viability using Cell Counting Kit-8 (CCK-8, Dojindo). For soft agar growth, cells were trypsinized and resuspended in RPMI 1640 media containing 0.4% agarose and 10% FBS. Transfected BE(2)-C and BE(2)-M17 cells were overlaid onto a bottom layer of solidified 0.8% agarose in RPMI 1640 media containing 10% FBS, at concentrations of 3×10^3^ cells per well in 6-well plates, and incubated for 3 weeks. Colonies were stained with 0.05% crystal violet, photographed, and quantified.

### Reverse transcription, semi-quantitative and quantitative real-time polymerase chain reaction

Total RNA was isolated using Trizol and reverse-transcribed to cDNA using High Capacity cDNA reverse transcription kit according to manufacturer's protocol (Applied Biosystems, Foster City, CA). Semi-quantitative PCR was performed using a Peltier Thermal Cycler (PTC-200) using specific 3'and 5' primers for *GLI1*: Forward 5'- CAGGGAGTGCAGCCAATACAG-3' and Reverse 5'- GAGCGGCGGCTGACAGTATA-3'.

The final product visualized on 1% agarose gel using a Gel Doc (Bio-Rad). QRT-PCR was performed using the Bio-Rad Thermocycler CFX96. SsoFAST EvaGreen Supermix, cDNA and specific 3' and 5' primers were incubated together using the manufacturer's protocol (Bio-Rad). GAPDH was used as control.

### Cell fractionation

Cells were harvested and the pellet was resuspended in hypotonic buffer (10 mM HEPES pH 7.9, 1.5 mM MgCl_2_, 10 mM KCl and protease inhibitors). After 20 min incubation on ice, cells were lysed using a 25-gauge needle. Cells were centrifuged for 10 min at 2000 rpm and supernatant was collected as the cytoplasmic fraction. The pellet was further washed with hypotonic buffer and resuspended in 1X cell lysis buffer. After sonication of resuspended pellet, the lysate was centrifuged for 10 min to obtain the nuclear fraction. α-tubulin and Lamin A/C were used as markers for cytoplasmic and nuclear fractions, respectively.

### Statistical analysis

Scoring index, relative luciferase activity were expressed as means ± SEM; statistical analyses were performed using student t-test for comparisons between the treatment groups. A *p* value of < 0.05 was considered significant.

## Supplementary Figures


