# Supplementary material for: Gli1 Transcriptional Activity is Negatively Regulated by AKT2 in Neuroblastoma
Source: Oncotarget. 2013 Jun 29;4(8):1149–57. doi: 10.18632/oncotarget.1074 (PMC3787147; doi:10.18632/oncotarget.1074)
Supplement: Supplementary file 1 [file oncotarget-04-1149-s001.pdf]

Gli1 Transcriptional Activity is Negatively Regulated by AKT2 in Neuroblastoma - Paul et al

Supplemental Materials and Methods

siRNA and Cyclopamine treatment

Human neuroblastoma cell lines, BE(2)-C, BE(2)-M17, SK-N-DZ, SK-N-AS, SK-N-SH, SK-N-BE(2), were purchased from ATCC. They were treated with siRNA (100nM) against non-targeting control (siNTC) or siGLI1 for 48 h or cyclopamine (0-20 µM; Tocris Bioscience, UK) for 72 h and assessed for cell survival by CCK-8.

Live-cell fluorescence imaging

BE(2)-C cells transfected with pEGFP or pEGFP-hGLI1 with or without myr-AKT2 were plated on glass coverslips and incubated for 24 h. The transfected cells were washed once with PBS and then viewed with a fluorescence microscope using a 20X objective lens. Cells from five different fields of view were counted and assessed for intracellular localization of GFP.

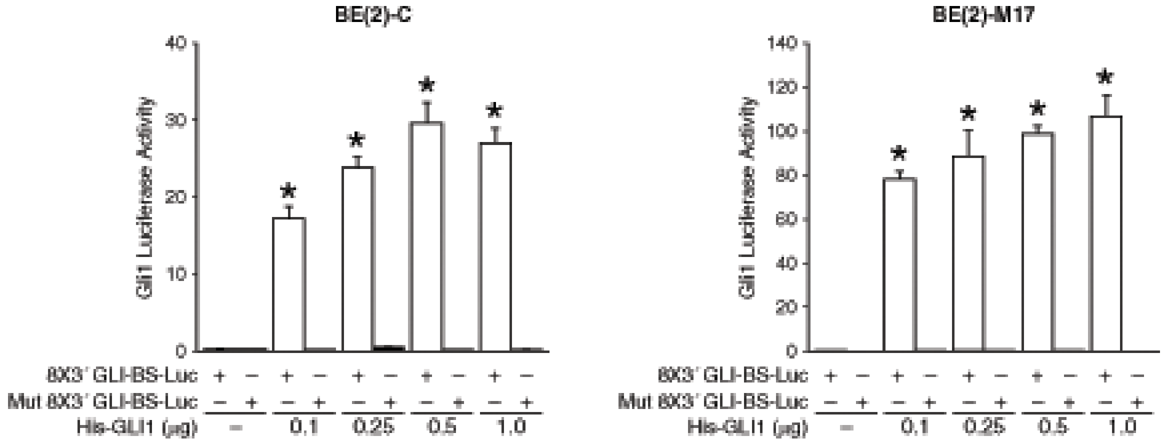

Figure S1: Dose-dependent increase in Gli1 luciferase activity. Human neuroblastoma cell lines, BE(2)-C and BE(2)-M17, were transfected with His-GLI1 at varying concentrations (0, 0.1, 0.25, 0.5 and 1 µg) and transcriptional activity was measured using 0.5 µg of Hh luciferase reporter (8x3' GLI-BS-Luc) or its mutant form (mut 8x3' GLI-BS-Luc). *Renilla* luciferase (20 ng) was used to normalize the values of Hh reporter luciferase (mean ± SEM; *=$p < 0.05$).

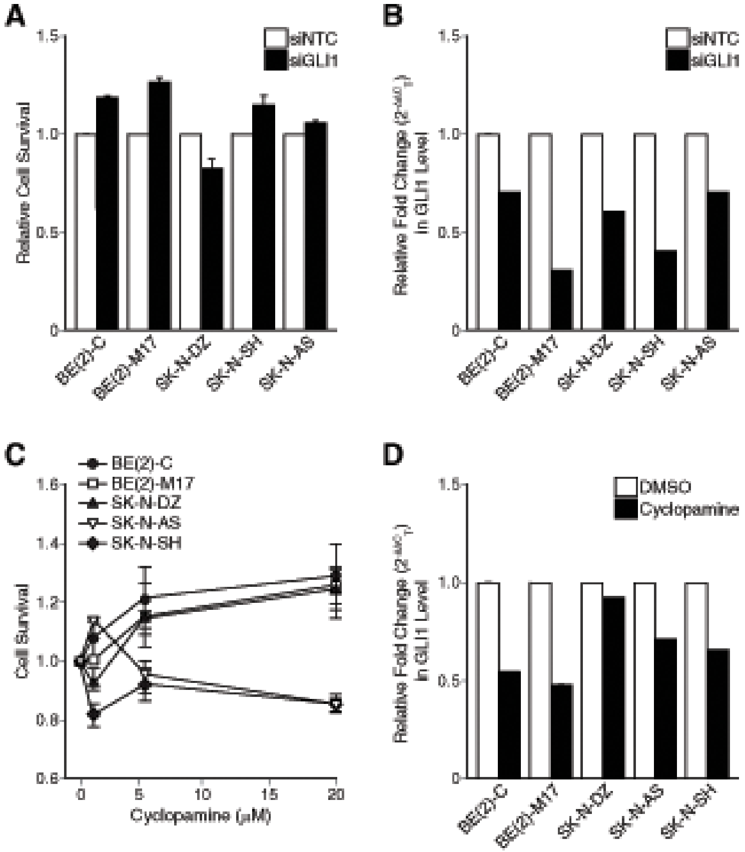

Figure S2: Targeting Hh signaling failed to inhibit neuroblastoma cell proliferation. (A) siRNA-mediated silencing of *GLI1* did not inhibit cell proliferation in neuroblastoma cells lines. (B) *GLI1* mRNA levels were checked by real-time PCR after transient transfection for 48 h. (C) Cyclopamine treatment (5-20 µM) failed to inhibit neuroblastoma cell proliferation. (D) *GLI1* mRNA levels were checked using real-time PCR after cyclopamine treatment (20 µM).

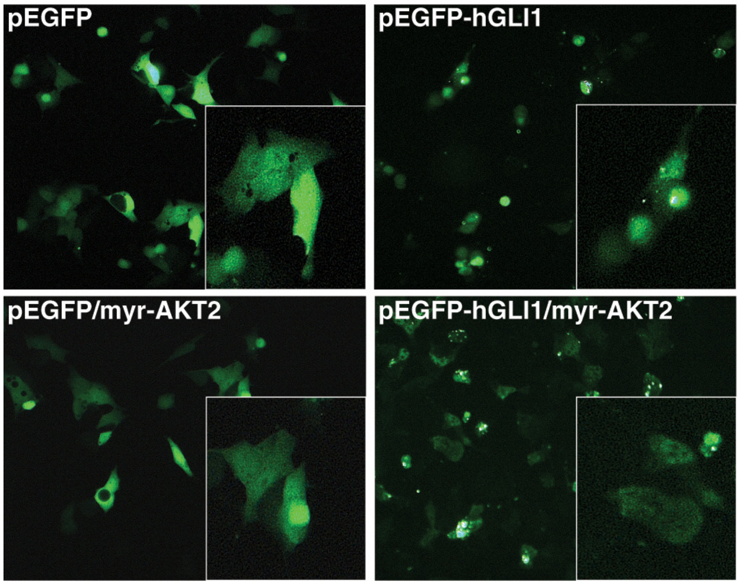

Figure S3: AKT2 decreased nuclear localization of Gli1. Gli1-GFP in pEGFP-hGLI1 transfected BE(2)-C cells was localized primarily in the nucleus, but undergo cytoplasmic shuttling upon AKT2 overexpression, as assessed by live-cell fluorescence imaging. There was no significant difference in the fluorescence localization in BE(2)-C cells transfected with pEGFP control vector with or without myr-AKT2 overexpression. Co-transfection of EGFP-hGLI1 and AKT2 markedly decreased the nuclear signal as observed in case of EGFP-hGLI1 alone.
